# [GADV]-Protein World Hypothesis on the Origin of Life

**DOI:** 10.1007/s11084-014-9383-4

**Published:** 2015-01-16

**Authors:** Kenji Ikehara

**Affiliations:** Nara Study Center, The Open University of Japan, Nara, 630-8589 Japan

**Keywords:** [GADV]-protein world hypothesis, GADV hypothesis, Origin of life, Pseudo-replication, protein 0^th^-order structure

## Abstract

RNA world hypothesis is widely accepted still now, as an idea by which the origin of life might be explained. But, there are many weak points in the hypothesis. In contrast, I have proposed a more reasonable [GADV]-protein world hypothesis or GADV hypothesis, suggesting that life originated from the protein world, which was formed by pseudo-replication of [GADV]-proteins. In this communication, I will discuss about the origin of life from the point of view of the GADV hypothesis.

Even though many researchers tried to solve the riddle of the origin of life, the question “Why the origin of life is still a mystery?” will remain unanswered until new, correct concepts are introduced. To this end, I have proposed the [GADV]-protein world hypothesis, or the GADV hypothesis in short, for the origin of life (Ikehara 2002; Ikehara 2005; Ikehara 2009; Ikehara 2012). The abbreviation GADV derives from four amino acids – Gly [G], Ala [A], Asp [D], and Val [V], − that formed the earliest proteins.

At least one new concept is introduced into the GADV hypothesis (Ikehara 2002; Ikehara 2005; Ikehara 2009; Ikehara 2012): the protein 0^th^-order structure or a specific amino acid composition will yield water-soluble globular proteins with a relatively high probability, even though amino acids are randomly joined. In other words, according to this concept, entirely new proteins could be produced at a high probability in the absence of any genetic function if proteins can be synthesized in the specific amino acid composition. One of protein 0^th^-order structures is [GADV]-amino acids (Fig. 1). The reason is because proteins composed of roughly equal amounts of [GADV]-amino acids satisfy the four conditions (hydropathy, α-helix, β-sheet, and turn/coil formabilities) for folding polypeptide chains into water-soluble globular structures, according to the analysis of database of extant microbial proteins, although it needs to confirm experimentally if such [GADV]-polypeptides are folded into water-soluble globular structures.

**Fig. 1:**
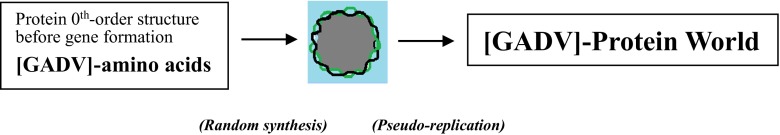
Water-soluble globular proteins would be produced at a high probability by random joining of [GADV]-amino acids. In GADV hypothesis, it is also assumed that [GADV]-protein world was formed by pseudo-replication of [GADV]-protein. Wavy lines indicate flexible surface structure or wobbling of surface amino acids of a water-soluble globular protein

It might be questioned if proteins containing about equal amounts of [GADV]-amino acids could be made before the establishment of the genetic system, since many kinds of organic compounds other than [GADV]-amino acids should be produced, and valine would be accumulated only at a much lower concentration than glycine, alanine and aspartic acid on the primitive Earth. These expectations are, of course, correct. But, I propose that [GADV]-amino acids would be selectively collected from various kinds of organic compounds through electrostatic interaction between negative and positive charges on carboxyl and amino groups of amino acids, respectively, and that peptides containing valine would preferentially assemble into [GADV]-proteins as aggregates through hydrophobic interaction among side chains of valine. [GADV]-based proteins could function efficiently to open the difficult pathway to the emergence of life, although problems on the ‘selective collection’ of [GADV]-amino acids and the ‘preferential assemblage’ of [GADV]-polypeptides are remained unsolved experimentally. Successively, GNC primeval genetic code and (GNC)_n_ genes would be invented, in order to compensate for the difficulties in joining directly the [GADV]-amino acids. Where G, C and N in GNC mean guanine, cytosine and either of four bases (G, C, A (adenine) and U (uracil)), respectively. So, GNC represents four genetic codes encoding four kinds of [GADV]-amino acids. It is supposed that the first life could emerge when the GNC code and (GNC)_n_ genes were casually invented.

In addition, the most important point for solving the riddle on the origin of life on the primitive Earth and/or in the Universe is to understand the processes how the genetic system, which is composed of genes, the genetic code and proteins, was created. To close this gap, I propose that life emerged from the [GADV]-protein world, in which nucleotides and oligonucleotides were synthesized, triggering the establishments of the GNC primeval genetic code and the formation of (GNC)_n_ genes.

The [GADV]-amino acids were synthesized on the primitive Earth and/or introduced from space. It is well known that those amino acids can be easily synthesized in Miller-type experiments and are contained in meteorites (van der Gulik, Massar et al. 2009). Next, [GADV]-oligopeptides and/or [GADV]-proteins as aggregates of these oligopeptides could be produced by repeated heat-drying processes of [GADV]-amino acids, for example in tide pools, and would be further accumulated by pseudo-replication to form [GADV]-protein world. The term “pseudo-replication” means a process where proteins comprising the same constituent set of amino acids (composition), which possess similar but different water-soluble globular structures, are generated by a random process without resorting to any genetic system. Subsequently, nucleotides and oligonucleotides were synthesized with the aid of catalytically active [GADV]-proteins. The accumulation of oligonucleotides triggered the generation of GNC primeval genetic code through stereospecific interaction between four GNC-containing oligonucletoides and four corresponding [GADV]-amino acids, or the dimerization of primitive tRNAs (Gly/Ala and Asp/Val) as Guimaraes has also reported (Guimaraes et al. 2008). Search or trial-and-errors for more efficient synthesis of [GADV]-proteins using the complexes than the direct synthesis among [GADV]-amino acids could assist establishing the GNC primeval genetic code, since the more efficient synthesis could step forward to the emergence of life, as a consequence. There is another problem on exhaustion of substrates, which might be faced with during formation of [GADV]-protein world. But, I conjecture that the formation process of [GADV]-protein world started, after [GADV]-amino acids accumulated sufficiently on the primitive Earth and the resulting [GADV]-proteins could synthesize [GADV]-amino acids long before the amino acids were exhausted.

Next, GNC-repeating sequences, or single-stranded (GNC)_n_ (ss-(GNC)_n_) genes, would be produced by phosphodiester bond formation between GNC codons or anticodons in the complexes. When the double-stranded (GNC)_n_ (ds-(GNC)_n_) genes were formed by synthesis of (GNC)_n_ sequence complementary to the single-stranded gene and replication system was established, the first life could emerge on the primitive Earth. Thus, search for more effective production of [GADV]-proteins made it possible to progress to the next stage, while always utilizing the protein 0^th^-order structure. It is also assumed that the sufficiently high catalytic activity of [GADV]-proteins functioned to form phosphodiester bond between nucleotides, to synthesize RNA or genes and to replicate the genes, although it should be also confirmed experimentally whether the assumptions are correct or not. I emphasize that a repeated heat-drying, or dehydration, process in tide pools on the primitive Earth would direct to the synthesis more strongly than to the degradation of the proteins. Therefore, one can conclude that the turning point from the inanimate matter to life happened upon the acquisition of ds-(GNC)_n_ genes and the emergence of replication system of genetic information.

As described above, the GADV hypothesis reasonably explains how the genetic system was created on the primitive Earth. In contrast, it is impossible to find a reasonable explanation for the establishment of the genetic code and creation of genes from the standpoint of the RNA world hypothesis. The reasons are as follows. In addition to frequently stressed weak points that it is quite difficult to synthesize nucleotides, oligonucleotides and RNA under prebiotic conditions, there are a number of other, probably fatal, weak points in the RNA world hypothesis. First, self-replication of RNA is principally impossible, because the replication requires RNA as a template without three-dimensional structure and simultaneously requires RNA with three-dimensional structure for catalytic function. Second, genetic information would never be formed from self-replicated RNA, since the genetic sequence has been always composed of triplet codons, which is never created by random joining of nucleotides one by one. Third, even if the genetic function were created with RNA, the function could not be expressed in the absence of the genetic code. This means that the genetic code must always precede the formation of genetic RNA. Forth, catalytic function of a three-dimensional RNA or ribozyme is never transferred to amino acid sequence of protein with the same function. So, I consider that life never emerged according to the RNA-world prebiotic scenario.

Nevertheless, it is usually assumed that life could never originate from a protein world, because active proteins could never be produced without genes. This argument follows from the diversity of possible sequences that even for small proteins composed of 100 amino acids would reach 20^100^ = ~10^130^, if 20 amino acids were joined randomly. In this diverse pool of sequences, active proteins would constitute a negligibly small fraction. In addition, they may also consider that RNA world hypothesis is the only idea for resolving the “chicken and egg relationship” between genes and proteins. These concerns, however, do not apply to the GADV hypothesis since proteins with weak catalytic activities could be produced with reasonable probability through direct random joining of [GADV]-amino acids in the absence of genetic function. This is possible because the diversity of sequences is smaller and they tend to fold into water-soluble globular structures, a prerequisite for catalytic activity. In addition, GADV hypothesis can explain how deal with the “chicken and egg relationship” on the primitive Earth. The relationship would be formed as going up from the lower ([GADV]-protein world) to the upper stream (genes) of the genetic flow in the present life system (Fig. 2), in the following order: (i) the formation of [GADV]-protein world, (ii) the establishment of the primeval GNC genetic code encoding [GADV]-amino acids, (iii) the formation of ss-(GNC)_n_ RNA gene, corresponding to mRNA in the modern genetic system, through joining of neighboring GNC codons or anticodons, and (iv) the formation of ds-(GNC)_n_ RNA gene, (5′-GNC-3′)_n_/(3′-CNG-5′)_n_, by synthesis of the complementary strand of the ss-RNA, followed by formation of ds-DNA gene.

**Fig. 2:**
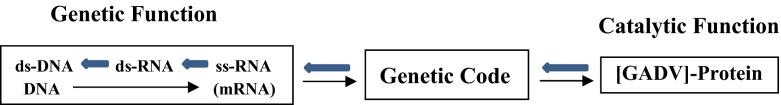
The “chicken and egg relationship” between genes and proteins was formed as going up (*thick blue arrows*) from the lower ([GADV]-protein world; catalytic function) to the upper stream (creation of genetic function) of the genetic flow. But, genetic information always flew from the upper to the lower stream (*thin black arrows*). ds-DNA, ds-RNA and ss-RNA mean double-stranded DNA, double-stranded RNA and single-stranded RNA, respectively

As described above, the GADV hypothesis has many strong points and can answer many questions on the origin of life. At this point, however, the only experimental result supporting this hypothesis is detection of enzymatic activities of [GADV]-peptides or [GADV]-proteins as aggregates for hydrolysis of a protein, bovine serum albumin (BSA) (Oba, Fukushima et al. 2005). Nevertheless, I emphasize that GADV hypothesis is not a purely theoretical idea, since the hypothesis is based on protein 0^th^-order structure such as [GADV]-amino acids, which satisfies the four structural conditions (hydropathy, α-helix, β-sheet and turn/coil formabilities), which were obtained from analysis of experimental data of microbial genes and proteins. Equally importantly, the assertion about activity of [GADV] proteins is testable in high throughput in vitro experiments. Such experiments are currently in progress.
